# Automated diagnosis of Sturge–Weber syndrome by detecting leptomeningeal angiomatosis from MRI using a multi-task deep learning framework

**DOI:** 10.3389/fnins.2026.1862520

**Published:** 2026-07-08

**Authors:** Jing Wu, Xindan Hu, Chenxi Xu, Chenghao Xue, Ruisheng Su, Yumei Yan, Ruolin Hou, Weiqun Bao, Tao Tan, Xiaoqiang Wang, Dake He, Lin Xu

**Affiliations:** 1 Department of Pediatric Neurology, Xinhua Hospital Affiliated to Shanghai Jiao Tong University School of Medicine, Shanghai, China; 2 School of Information Science and Technology, ShanghaiTech University, Shanghai, China; 3 Department of Biomedical Engineering, Eindhoven University of Technology, Eindhoven, Netherlands; 4 Faculty of Applied Science, Macao Polytechnic University, Macau, Macao SAR, China; 5 Department of Pediatric Neurosurgery, Xinhua Hospital Affiliated to Shanghai Jiao Tong University School of Medicine, Shanghai, China; 6 State Key Laboratory of Advanced Medical Materials and Devices, Shanghai, China

**Keywords:** leptomeningeal angiomatosis, magnetic resonance imaging, multi-scale harmonization, multi-task learning, Sturge–Weber syndrome

## Abstract

**Objectives:**

Sturge–Weber Syndrome (SWS) is a rare neurocutaneous disorder requiring early and accurate diagnosis. This study aims to develop a deep learning framework for automatic diagnosis of SWS by detecting leptomeningeal angiomatosis (LA) in brain magnetic resonance images (MRI).

**Methods:**

This retrospective study includes 40 SWS patients and 101 healthy controls. T1-weighted MRI were collected over 15 years with different scanners. Multi-scale harmonization was proposed to normalize the images to uniform mean and standard deviation. We developed a deep learning model based on the UNet framework with convolutional neural networks and transformer. A multi-task pipeline was designed to perform LA segmentation and SWS classification. The model was pre-trained on a public dataset and fine-tuned and tested on our dataset using a 5-fold cross validation. The segmentation and classification results were compared with the ground truth and human readers using various metrics at the voxel and LA level.

**Results:**

For LA segmentation, our model achieved overall Dice, voxel-level sensitivity, specificity, accuracy, and kappa of 0.768, 0.772, 0.992, 0.983, and 0.760, respectively. The LA volume segmented by our model showed a high agreement and consistency with the ground truth, outperforming the human readers. For SWS classification, our model achieved sensitivity, specificity, accuracy, and AUC of 0.974, 1.000, 0.993, and 0.998, respectively.

**Conclusion:**

We developed a multi-task learning framework for automated LA segmentation and SWS classification. This single-center proof-of-concept study shows promising performance in both tasks. External validation may boost the clinical application of the developed model, assisting less-experienced neurologists for improved SWS diagnosis.

## 1 Introduction

Sturge–Weber Syndrome (SWS) is a rare neurocutaneous disorder with an estimated occurrence rate between 1 to 2.5 per 50,000 live births (Comi, 2007). Early diagnosis of SWS is essential for proper medical intervention in order to prevent the development of further neurological complications (Pascual-Castroviejo et al., 2008; Maria et al., 1998; Jagtap et al., 2013), such as seizures, hemiparesis, hemianopia, vascular headache, and intellectual disability (Sudarsanam and Ardern-Holmes, 2014; Sujansky and Conradi, 1995; Fogarasi et al., 2013).

Intracranial vascular anomaly, known as leptomeningeal angiomatosis (LA), is suggested as the clinical manifestation of SWS (Schirmer, 1860; Sturge, 1969; Bodensteiner and Roach, 1999). Magnetic resonance imaging (MRI), particularly T1-weighted MRI, has been most frequently employed for LA detection and thus SWS diagnosis (Bar et al., 2019; Mengistie et al., 2025). However, detection of LA in MRI images is currently carried out by visual inspection of neuro-radiologists. This is time-consuming and experience-demanding, resulting in low inter-rater reliability and sometimes even missed diagnosis. An automatic tool supporting the detection of LA in MRI images is therefore demanded for improved diagnosis of SWS.

Machine-learning or deep-learning based computer-aided diagnosis has been proposed for automated tumor detection, classification, and segmentation (Doi, 2007). Particularly, convolutional neural networks (CNNs) show promising performance in feature extraction and tumor classification (Liu et al., 2019). UNet and its variants (Ronneberger et al., 2015; Çiçek et al., 2016; Isensee et al., 2020) have achieved great success in tumor segmentation on public medical datasets such as BraTs (Menze et al., 2015; Bakas et al., 2017). However, these methods have never been developed for automated SWS diagnosis. The main challenges of applying deep learning models to MRI-based SWS diagnosis are the small size and uneven distribution of the datasets due to the rarity of the disease. Besides, the MRI data may originate from different scanners as data collection may span more than dozens of years.

The aim of the present study is therefore to develop a deep learning method for automatic SWS diagnosis based on T1-weighted MRI images. To this end, we have developed a deep learning model by embedding the Transformer model (Dosovitskiy et al., 2021) into an nnU-Net framework (Isensee et al., 2020). We propose a multi-scale harmonization technique prior to the deep learning models in order to normalize the MRI images collected in 15 years with various equipment and magnetic-field strengths. 3D convolution and Transformer are then employed to promote the extraction of spatial features and improve 3D spatial context representation. A multi-task pipeline is developed to perform the segmentation of LA and the classification between SWS patients and healthy subjects. In addition, to overcome the challenge of inadequate data, the proposed model is first pre-trained on a public brain MRI dataset and subsequently fine-tuned on our specific SWS dataset collected in clinical practice.

The main contributions of this study are summarized as follows:

- We propose a unified multi-task framework for simultaneous leptomeningeal angiomatosis segmentation and SWS classification, enabling end-to-end automated diagnosis from MRI data.
- We introduce a multi-scale harmonization strategy to address domain shifts caused by multi-scanner and long-term clinical data acquisition, improving model robustness and generalization.
- We integrate CNN and Transformer modules within the nnU-Net architecture to capture both local spatial features and global contextual dependencies in 3D MRI data.
- We validate the proposed method using a single-center clinical dataset and demonstrate superior performance compared with state-of-the-art models and human readers, highlighting its potential for real-world clinical application.

## 2 Methodology

**Figure 1:**
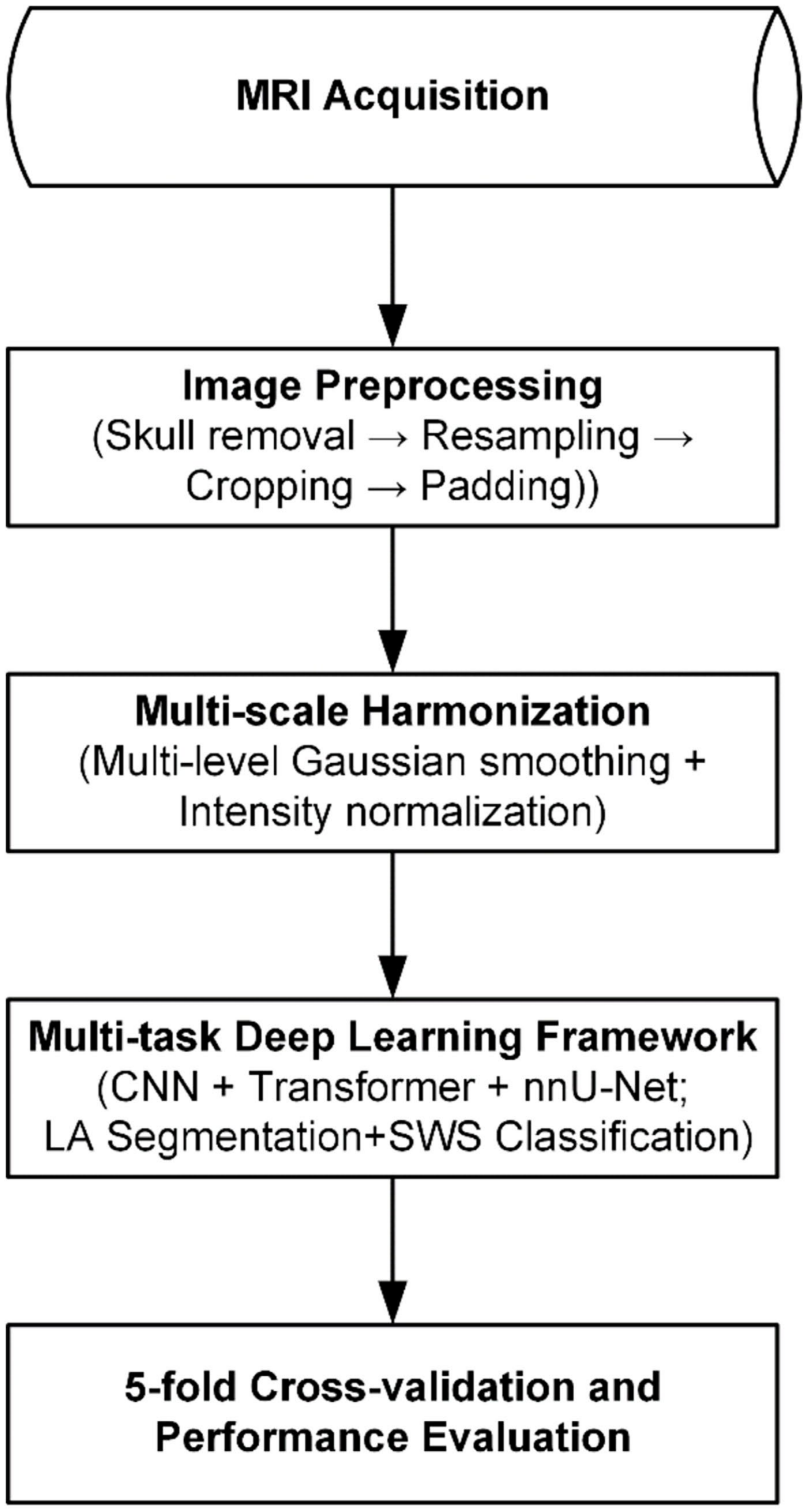
Overall workflow of the proposed study.

The overall workflow of the proposed study is illustrated in Figure 1. T1-weighted MRI scans from SWS patients and healthy controls were collected and preprocessed through skull removal, resampling, cropping, and zero-padding. To reduce inter-scanner variability caused by multi-center and long-term data acquisition, a multi-scale harmonization strategy was applied to normalize image intensity distributions. Subsequently, the preprocessed and harmonized images were jointly used as inputs to a multi-task deep learning framework that integrates CNN and Transformer modules within an nnU-Net architecture. The framework simultaneously performs LA segmentation and SWS classification by exploiting shared feature representations. Finally, the model was pre-trained on the BraTS2021 dataset, fine-tuned on the SWS dataset using a five-fold cross-validation strategy, and evaluated using quantitative metrics for both segmentation and classification performance.

### 2.1 Participants and datasets

This study was approved by the Ethics Committee at Xinhua Hospital Affiliated to Shanghai Jiao Tong University School of Medicine with the number XHEC-D-2024-090. Written informed consent of each subject was waived by the ethics committee as it was designed as a retrospective study.

Patients diagnosed as SWS at Xinhua Hospital Affiliated to Shanghai Jiao Tong University School of Medicine between June 2008 and August 2023 were recruited. Patient exclusion criteria include: (a) younger than 3 months; (b) syndrome type II, i.e., without LA; (c) missing clinical information or poor-quality images preventing manual annotation. Consequently, 40 SWS patients were involved in this study. In addition, 101 healthy subjects were considered as healthy controls (HC).

T1-weighted MRI images were acquired using different scanners, including Siemens, Philips, General Electric, and United Imaging, with two different magnetic field strengths, i.e., 1.5 T and 3 T. Details of the patient information and the MRI parameters are listed in Table 1. For the MRI image of each patient, the ground truth (GT) LA was labeled by an experienced neuro-radiologist from Xinhua Hospital Affiliated to Shanghai Jiao Tong University School of Medicine. However, marking the abnormal intracranial vessels is challenging. Instead, the neuro-radiologist marked the brain areas containing LA using the well-known ITK-SNAP toolbox (Yushkevich et al., 2006).

**Table 1:** Patient information and MRI scanners and parameters.

Scanners	SWS	HC	TR/TE (ms)	Field of view (mm^2^)	Matrix size (pixel^2^)	Slice thickness
Philips (3 T)	5	71	1800/20	230 × 187	260 × 189	5 mm
General Electric (3 T)	17	3	1750/24	200 × 200	320 × 224	5 mm
General Electric (1.5 T)	15	7	520/9.9	240 × 240	320 × 160	5 mm
Siemens (3 T)	0	6	2000/36	200 × 200	320 × 224	5 mm
Siemens (1.5 T)	3	13	1600/8.9	200 × 200	256 × 180	5 mm
United Imaging (3 T)	0	1	2215/12.04	160 × 160	352 × 264	4 mm
Patient information						
Total	40	101				
Girls: boys	13:27	33:68				
Age (month)	5–696, IQR = 70	4–96, IQR = 29				

Furthermore, in each MRI image, the skull was first removed using the FSL toolbox (Smith et al., 2004; Woolrich et al., 2009; Jenkinson et al., 2012) as it may confuse the identification of contrast-enhanced LA. Then the images recorded with different scanners and protocols were resampled to uniform physical spacing of 0.47×0.47×6.5 mm^3^. In addition, each image underwent cropping to contain only the brain area. However, due to different brain sizes among different subjects, all cropped images were zero-padded to a unique size, i.e., 320 pixels × 320 pixels, determined by the largest size of the cropped image, in order to keep consistency for further analysis.

### 2.2 Multi-scale harmonization strategy

**Figure 2:**
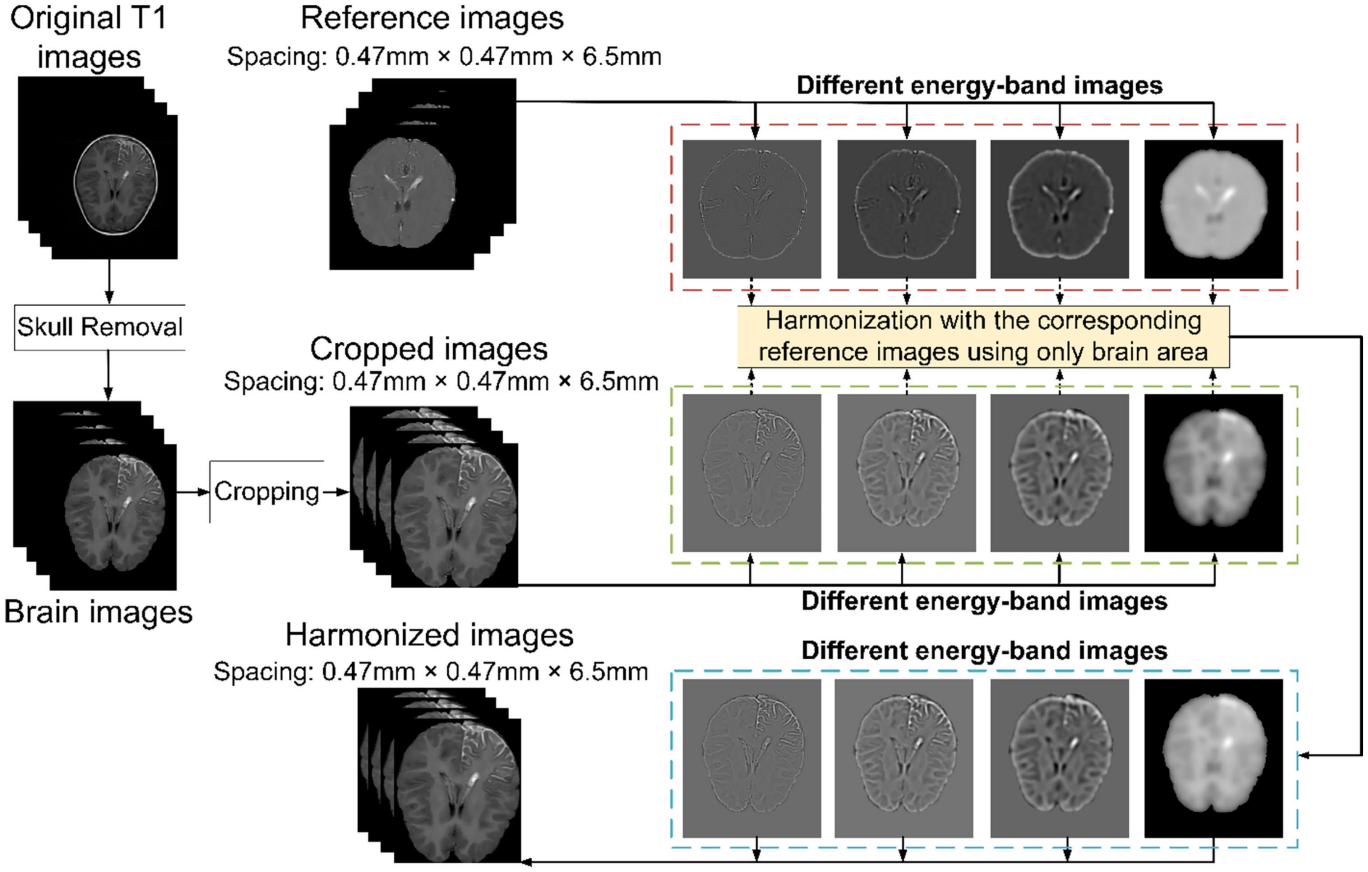
Data preprocessing and harmonization.

A multi-scale harmonization technique (GE Precision Healthcare LLC, 2023) was proposed to address domain shifts caused by multi-scanner MRI acquisition over long-term clinical collection. As shown in Figure 2, three levels of Gaussian smoothing were applied to the brain regions of each image, generating, together with the original image, four images with different energy bands:


$$Y_{n}=G_{k_{n}}*X,$$

where *X* is the input image, $G_{k_{n}}$ represents the $n^{\mathrm{th}}$ Gaussian kernel with kernel size $k_{n}=0,2,4,6$ for n=0,1,2,3 . Particularly, $k_{n}=0$ indicates the original image without smoothing. The mean and standard deviation of each energy-band image were then normalized to those extracted from a high-quality reference image (pre-selected) undergoing the same Gaussian-smoothing procedure, expressed as


$${\tilde{Y}}_{n}=\frac{Y_{n}-\mu_{n}}{\sigma_{n}}\sigma_{n\_\mathrm{ref}}+\mu_{n\_\mathrm{ref}},$$

where μ and σ indicates mean and standard deviation, respectively, and $\mu_{n\_\mathrm{ref}}$ and $\sigma_{n\_\mathrm{ref}}$ are calculated from the smoothed reference image $Y_{n\_\mathrm{ref}}$ computed as $Y_{n\_\mathrm{ref}}=G_{k_{n}}*X_{\mathrm{ref}}$ . The normalized four-channel images were then combined into a single image to achieve an overall harmonization, i.e., $Y_{\text{harm}}=\sum_{n=0}^{3}{\tilde{Y}}_{n}$ .

### 2.3 Multi-task deep learning framework

**Figure 3:**
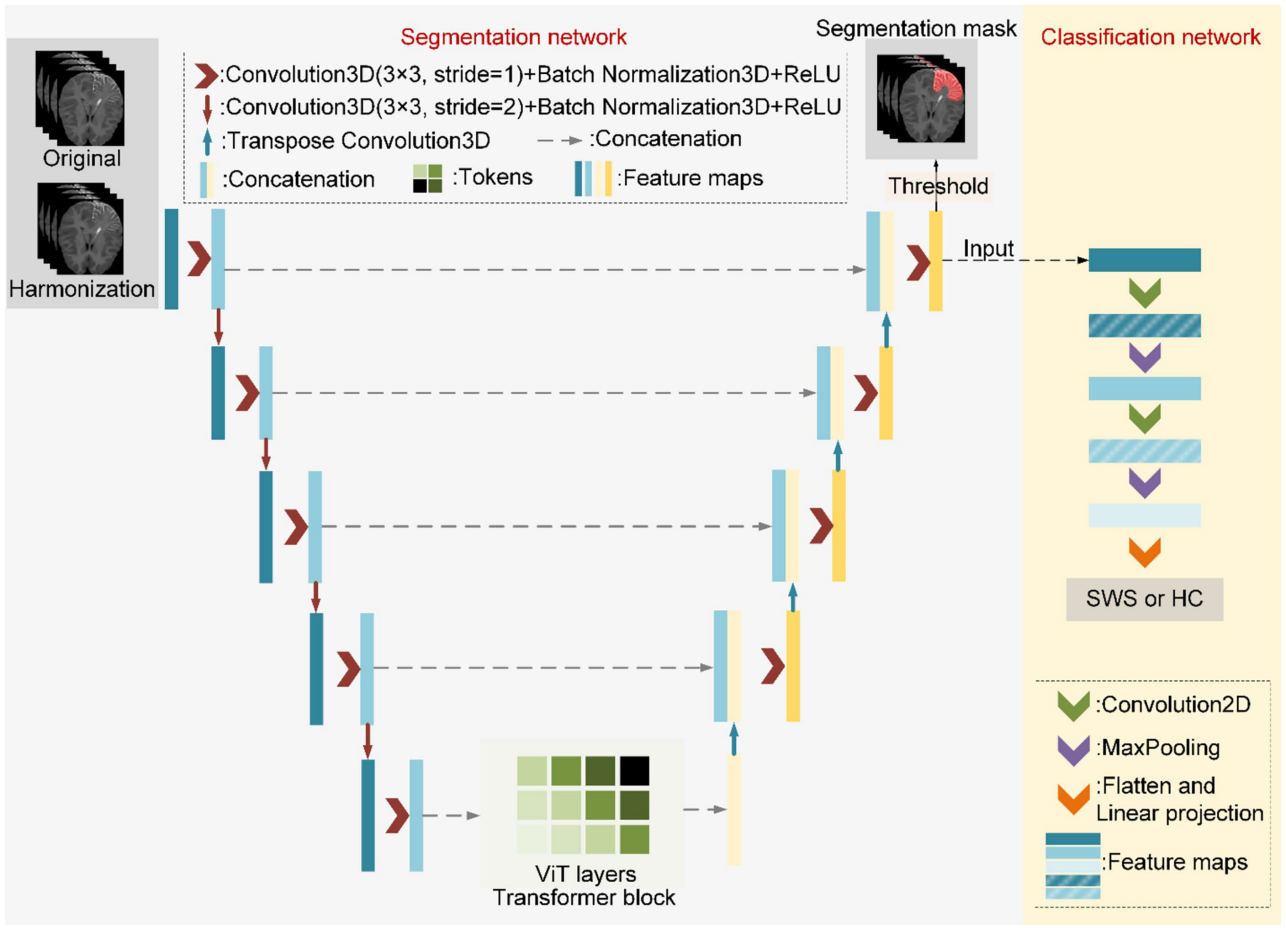
The architecture of the proposed deep learning model. The model consists of a segmentation network and a classification network. The last decoding layer of the segmentation network is the input of the classification network. For the segmentation network, we combined the CNNs and Transformer in the nnU-Net framework, and employed the combination of Dice loss and cross entropy as the loss function. A deep supervision was performed by calculating the combined loss function at each decoding level to achieve better segmentation of LA. For the classification network, a 2D CNN was used and the cross entropy was employed as the loss function.

In this study, we developed a multi-task learning strategy to jointly perform lesion segmentation and disease classification. The overall architecture of the proposed framework is shown in Figure 3.

#### 2.3.1 Segmentation network

The segmentation network was designed for LA identification from multi-channel T1 MRI images. It was achieved by combining 3D CNNs and Transformers in an nnU-Net framework. 3D CNN is ideal to process sequential images due to its capability to extract and map slice-to-slice and voxel-to-voxel features (Çiçek et al., 2016; Chen et al., 2019; Wang et al., 2021). As shown in Figure 3 (left part), our model was built based on the UNet framework, particularly nnU-Net (Isensee et al., 2020), with input images consisting of both cropped images and harmonized images. The two input images were concatenated as a 2-channel input and processed jointly by one encoder.

The UNet framework consisted of six encoding layers and the corresponding decoding layers. In the encoding branch, each encoding layer consisted of a 3D CNN followed by a batch normalization (Ioffe and Szegedy, 2015) and ReLU (Nair and Hinton, 2010) activation. Max-pooling was performed after each encoding layer in order to eliminate unnecessary information, enabling the extraction of multi-scale features from the input images.

The backbone of the decoding layers was the same as the encoding layers. The feature map of each encoding layer was concatenated to that of the corresponding decoding layer in order to enhance the segmentation with low-level characteristics from shallow convolution. Different from the traditional nnU-Net, Transformer blocks (Dosovitskiy et al., 2021) were introduced at the bottom of the UNet framework to improve feature fusion and global attention, particularly suitable for 3D MRI image analysis with unshaped LA (Zhao et al., 2020; Ji et al., 2023). Specifically, the self-attention mechanism can be formulated as:


$$\text{Attention}(Q,K,V)=\text{softmax}(\frac{QK^{T}}{\sqrt{d_{k}}})V,$$

where Q , K , and V denote the query, key, and value matrices, respectively, and $d_{k}$ is the dimensionality of the key.

In our supervised training process, the combination of cross-entropy loss and Dice loss was employed to measure the discrepancy between the predicted segmentation and the ground truth. These two loss functions are defined as follows:


$$L_{\mathrm{CE}}=-\frac{1}{N}\sum_{i=1}^{N}[y_{i}\log(p_{i})+(1-y_{i})\log(1-p_{i})],$$

$$L_{\text{Dice}}=1-\frac{2\sum_{i=1}^{N}p_{i}y_{i}+\varepsilon }{\sum_{i=1}^{N}p_{i}+\sum_{i=1}^{N}y_{i}+\varepsilon },$$

where $p_{i}\;$ denotes the predicted probability for the target class at pixel i , $y_{i}$ represents the corresponding ground truth, N is the total number of pixels, and ε is a small constant to avoid division by zero. The overall loss function is then defined as:


$$L_{\text{total}}=\frac{1}{2}L_{\mathrm{CE}}+\frac{1}{2}L_{\text{Dice}}.$$

To further improve the segmentation performance, a deep-supervision strategy was employed by introducing auxiliary losses at each decoding layer with exponentially decayed weights:


$$L=\sum_{i=1}^{M}w_{i}L_{\text{total}}^{i},w_{i}=\frac{1}{2^{i}},$$

where i denotes the index of the decoder layer and M is the total number of supervised layers.

#### 2.3.2 Classification network

In the present study, another task is to distinguish between SWS patients and HC. Using the feature maps of the last decoding layer of the segmentation network as inputs, another CNN network was developed for the classification task, as shown in Figure 3 (right part). The classification network consisted of two layers of 2D convolution with max-pooling and a multilayer perceptron.

For the classification task, cross-entropy loss was adopted to supervise the network training, defined as:


$$L_{\mathrm{CE}}^{\mathrm{cls}}=-\sum_{c=1}^{C}y_{c}\log(p_{c}),$$

where C denotes the number of classes, $y_{c}$ is the ground truth label for class C , and $p_{c}$ represents the predicted probability for class C .

### 2.4 Training strategy and implementation

Due to the limited subject size and the lack of external SWS dataset, the proposed segmentation model was first pre-trained on a public dataset, i.e., BraTs2021 (Baid et al., 2021), for 1,000 epochs. The BraTs2021 dataset consists of 2000 MRI scans of brain gliomas acquired from different institutions under standard clinical conditions. Although the gliomas tumors are biologically different from the LA, the BraTs dataset provides rich representations of anatomical structures and lesion characteristics in 3D brain MRI. Instead of assuming biological equivalence between gliomas and LA, BraTS was used solely to initialize generic MRI feature representations before fine-tuning on the SWS dataset.

For LA segmentation, the pre-trained model was fine-tuned and tested on our 40 SWS dataset. In order to optimize the usage of the available data, we utilized a 5-fold cross-validation strategy. The data was partitioned into five non-overlapping subsets, with four subsets were used for training and the remaining subset for testing. By rotating this process five times, each subset was utilized once for testing, and the results were averaged to generate a robust performance estimate. All images from a single subject were kept in the same fold to prevent subject-level leakage.

For SWS classification, the pre-trained model was fine-tuned and tested on our dataset consisting of 40 SWS patients and 101 HC using similar 5-fold cross-validation. The patients and HC were independently split to five folds, and the training set contained 4-fold patient data and 4-fold HC. In each rotation, three augmentation techniques, including contrast adjustment (Agarwal et al., 2024), intensity shift (Garcea et al., 2023), and addition of Rician (Khaleel et al., 2018) and/or Gaussian noise (Li et al., 2021), were performed to handle the imbalanced data between the SWS patients and HC. Specifically, 13 intensity levels (−60 to 60 in steps of 10) and 3 contrast levels (0.8, 0.9, 1.2) were considered for the SWS patients, expanding the patient data by a factor of 40 ( 3×13+1 ). For HC, 5 intensity levels (−60 to 60) and 3 contrast levels (0.8, 0.9, 1.2) were considered to expand the data by a factor of 16 ( 3×5+1 ). Besides, Rician and Gaussian noise were added to the intensity-and-contrast shifted images with a probability of 0.5 and 0.7, respectively. Note that the augmentation was restricted to the training set to avoid information leakage.

The deep learning model was implemented based on the Pytorch framework with the Python version of 3.9. The model was trained on a NVIDIA GeForce RTX 3090 graphic processing unit with 24 GB. The number of epochs and batch size were 250 and 8, respectively. The Stochastic Gradient Descent was used as an optimizer to train the model with an initial learning rate (LR) of 0.01, a weight-decay value of 0.00003 and a momentum value of 0.99 (Sinha and Griscik, 1971). The learning rate was dynamically updated in each epoch, given as


$${\mathrm{LR}}_{k}={\mathrm{LR}}_{1}\times {\left(1-\frac{k}{M}\right)}^{0.9},$$

where M=250 was the maximum epoch number, k was the number of current epoch, and ${\mathrm{LR}}_{1}$ was the initial LR.

Notably, both training and inference were performed in a patch-based strategy. Random patch sampling was used during training, and sliding-window prediction was used during testing. The patch-wise predictions were subsequently merged using Gaussian-weighted aggregation to generate the final segmentation output. This technique has been commonly employed in the field of computer vision and has proven effective in optimizing image analysis and recognition tasks (Isensee et al., 2020; Verelst and Tuytelaars, 2022; Chen et al., 2023).

### 2.5 Statistical analysis

For LA segmentation, we calculated the Dice score (DS) to quantify the agreement between the final segmentation of the model and the ground truth. Besides, sensitivity (ST), specificity (SP), accuracy (ACC), area under the receiver operating characteristic curve (AUC), kappa coefficient (K), volume similarity (VS; Reddy et al., 2013), and averaged Hausdorff distance (HD; Müller et al., 2022) were calculated to assess the consistency of the segmentation with the ground truth at voxel level.

We calculated also the agreement, indicated by Pearson coefficient, and consistency, indicated by concordance correlation coefficient (CCC), between the LA volume derived from the segmentation model and that of the ground truth, and plotted the similarity and Bland–Altman figures (Giavarina, 2015). In addition, two extra radiologists annotated the brain areas containing LA using the ITK-SNAP toolbox (Yushkevich et al., 2006), and the agreement and consistency in LA volume between each of the extra radiologists and the ground truth were assessed in similar ways.

For SWS classification, a confusion matrix was obtained by the 5-fold cross validation, from which ACC, ST, and SP were calculated to examine the classification performance of the model. Besides, we plotted the receiver operating characteristic curve and calculated the AUC to assess the classification power of our model.

## 3 Results

The dataset for LA segmentation included 40 SWS patients (female 13), and the dataset for SWS classification consisted of the same 40 SWS patients and 101 HC (female 33). The age of the patient cohort was between 5 and 696 months, with median and interquartile range (IQR) of 24 and 70 months, respectively. For the HC, the age was between 2 and 96 months, with median and IQR of 17 and 29 months, respectively. The average LA volume of the SWS patients was 96,803 mm^3^ (std 69,735 mm^3^). A global trend of increased Dice score with increased lesion size was observed in our results with a Person correlation coefficient of 0.49 and a *p*-value of 0.0018.

### 3.1 LA segmentation

**Figure 4:**
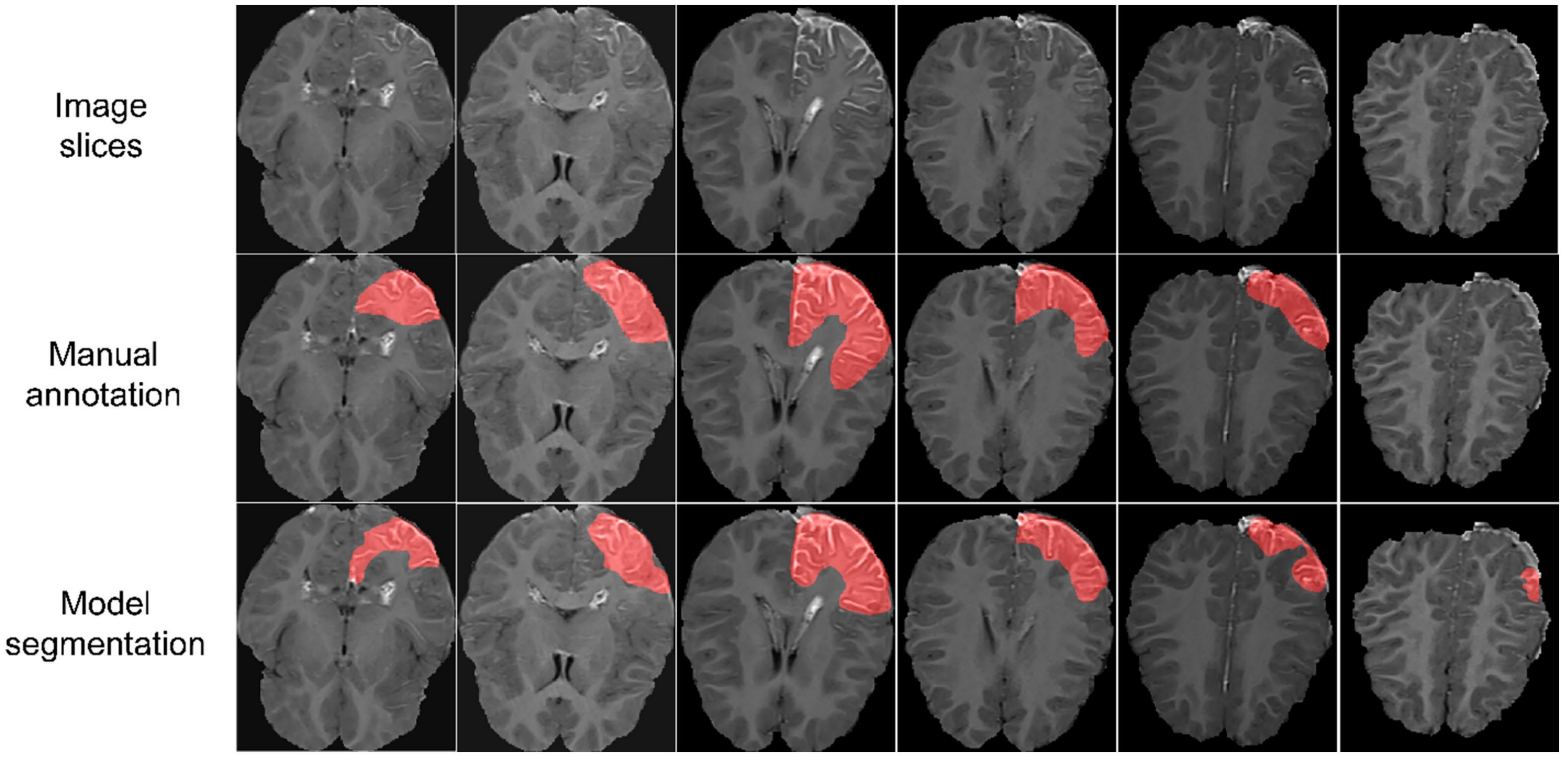
Examples of the pre-processed images (first row), the ground truth annotated by the experienced neuro-radiologist (row 2), and the model segmentation (row 3).

Figure 4 shows a representative example of LA segmentation achieved by our model. It is clear that the LA areas identified by our model are quite similar to that of the ground truth. The overall mean Dice coefficients were 0.768 (95% CI: 0.721 ~ 0.816) and median of 0.820. The voxel-level metrics achieved by our model were: ST of 0.772 (95% CI: 0.725 ~ 0.820), SP of 0.992 (95% CI: 0.990 ~ 0.994), ACC of 0.983 (95% CI: 0.980 ~ 0.986), AUC of 0.971 (95% CI: 0.956 ~ 0.987), K of 0.760 (95% CI: 0.713 ~ 0.807), and averaged HD of 38.02 (95% CI: 28.14 ~ 47.90). Besides, to demonstrate the superiority of our model, the segmentation performance of our model was compared to the traditional 3D UNet (Çiçek et al., 2016) and nnU-Net (Isensee et al., 2020), as reported in Table 2. Our model outperforms those two models in most of the adopted quantitative metrics.

**Table 2:** Segmentation results of the proposed model, the two UNet models, and the two human readers.

Method	DS	ST	SP	AUC	ACC	K	VS	HD (mm)
3D U-Net	0.477 (0.400–0.555)	0.686 (0.597–0.775)	0.940 (0.923–0.956)	0.734 (0.692–0.775)	0.933 (0.918–0.948)	0.449 (0.373–0.526)	0.638 (0.559–0.717)	97.6 (82.4–112.8)
nnU-Net	0.768 (0.724–0.812)	0.773 (0.727–0.819)	0.992 (0.990–0.994)	0.972 (0.957–0.986)	0.982 (0.979–0.985)	0.759 (0.715–0.802)	0.897 (0.867–0.927)	42.6 (29.4–55.8)
Proposed	**0.768 (0.721–0.816)**	0.772 (0.725–0.820)	**0.992 (0.990–0.994)**	0.971 (0.956–0.987)	0.983 (0.980–0.986)	**0.759 (0.713–0.807)**	**0.918 (0.888–0.948)**	**38.0 (28.1–47.9)**
Reader1	0.629 (0.565–0.694)	**0.824 (0.777–0.870)**	0.989 (0.986–0.992)	/	0.987 (0.984–0.990)	0.624 (0.560–0.688)	0.736 (0.664–0.809)	56.6 (40.9–72.4)
Reader2	0.658 (0.600–0.716)	0.794 (0.748–0.840)	0.991 (0.988–0.995)	/	**0.989 (0.985–0.992)**	0.653 (0.595–0.711)	0.793 (0.725–0.862)	47.0 (35.5–58.5)

Bold values indicate the best performance.

**Figure 5:**
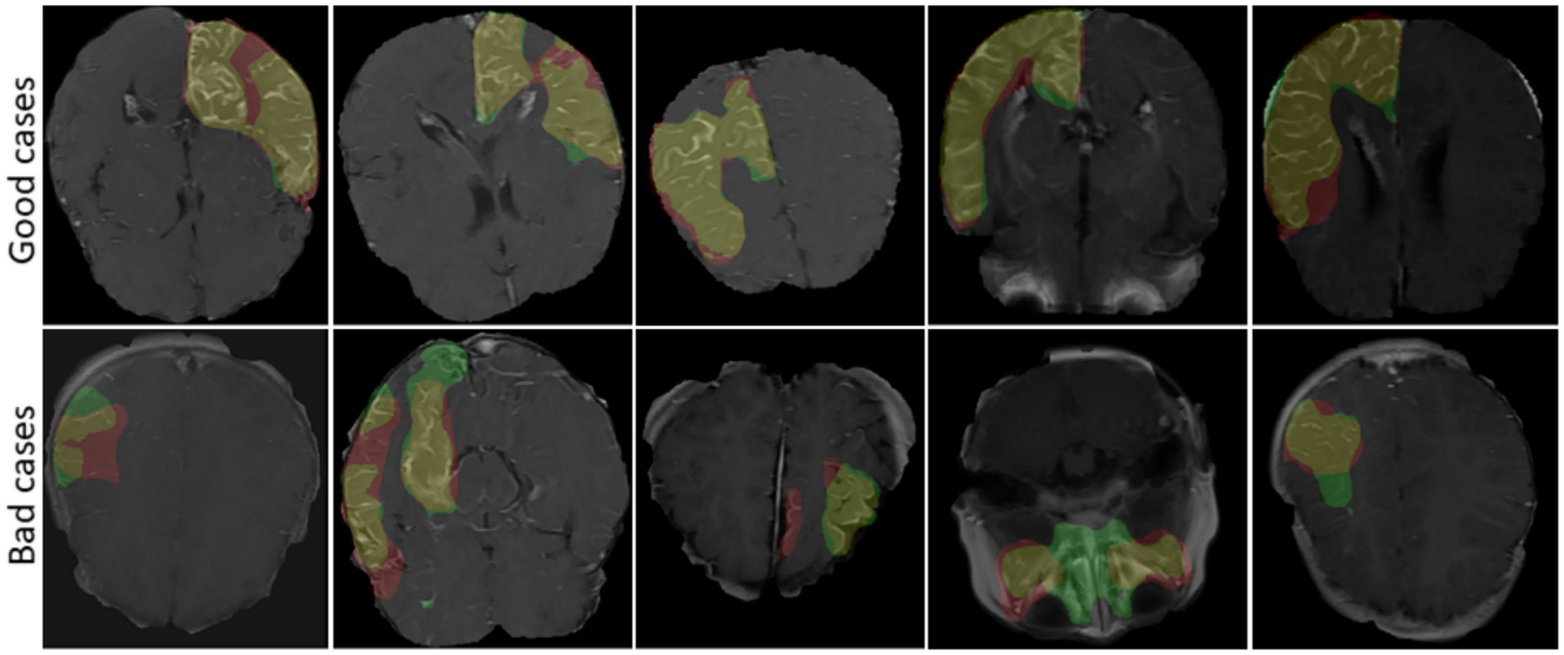
Examples of good- and bad-cases in LA segmentation. Red: ground truth; green: model segmentation; orange: overlap.

To future understanding the model’s strength and failure, examples of the best- and worst-cases are illustrated in Figure 5. In the best-cases, our model can successfully capture the LA region, producing well segment results as accurate as the ground truth. However, in the worst-cases, the model may miss some LA regions, such as small isolated regions and regions with limited vessel enhancement.

### 3.2 SWS classification

**Figure 6:**
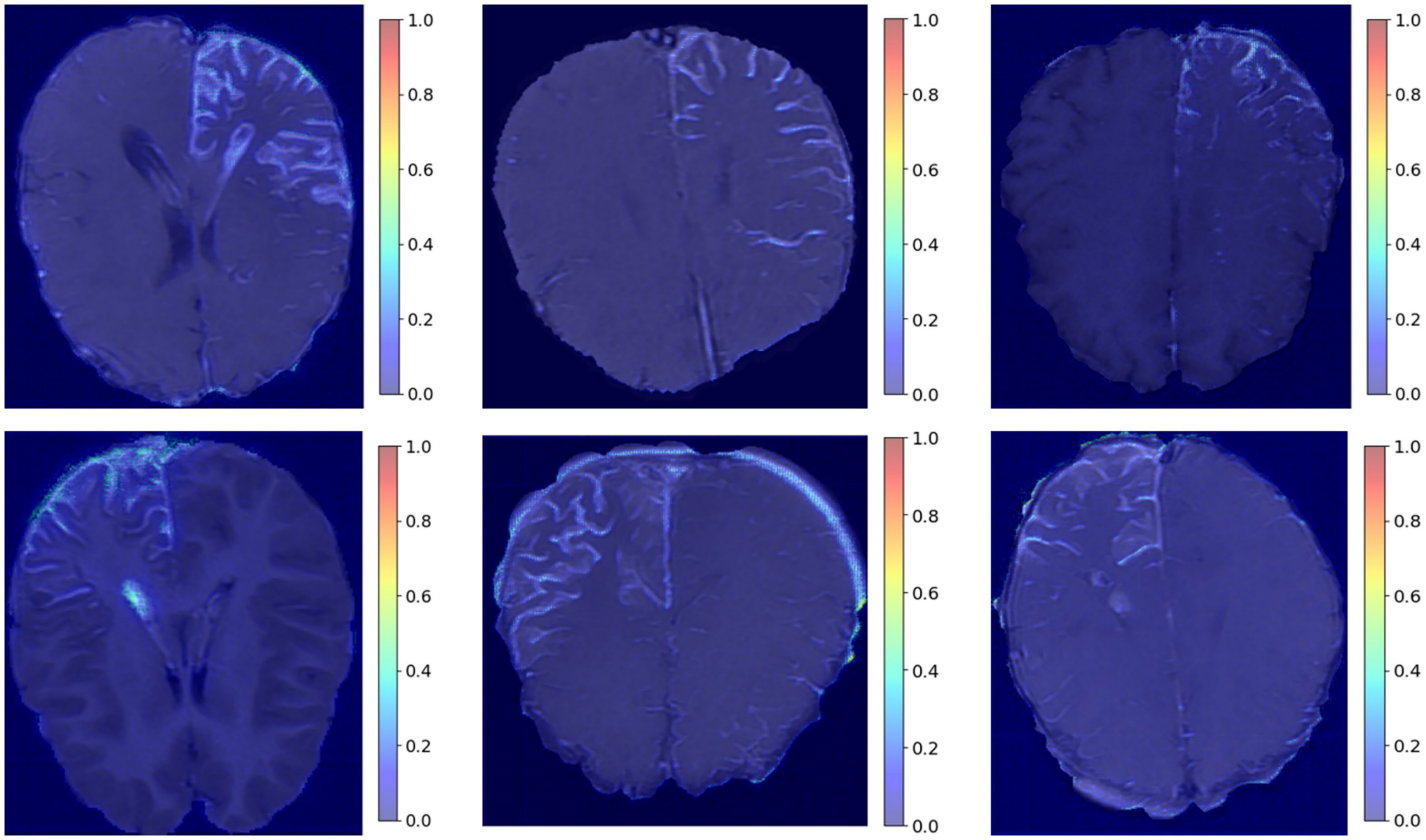
Examples of feature activation maps for effective SWS classification.

The segmentation results are used as the input of the classification model in order to distinguish between HC and SWS patients. Our model achieves excellent classification performance, i.e., sensitivity of 0.974, specificity of 1.000, accuracy of 0.993, and AUC of 0.998. The feature activation map showing in Figure 6 highlights the image regions influencing the model’s classification decision, which indeed correspond to the LA regions identified by the segmentation network. Quite similar results are observed when using the nnU-Net to generate the segmentation results. However, the traditional 3D-UNet is not considered to generate the segmentation results for classification as its performance in segmentation is much worse than that of the nnU-Net and our proposed model.

### 3.3 Ablation studies

Ablation study was performed in order to demonstrate the effectiveness of the proposed harmonization (employed for both segmentation and classification) technique as well as the data augmentation strategy (employed only for classification). The ablation results on harmonization for LA segmentation is reported in Table 3. It is clear that the harmonization technique yields improved segmentation performance in all the adopted quantitative metrics. Furthermore, the ablation results on both harmonization and data augmentation for SWS classification is reported in Table 4. The proposed model integrating harmonization and data augmentation achieves the best classification performance. These results demonstrate the effectiveness of the proposed harmonization and data augmentation strategy.

**Table 3:** Ablation results of the harmonization strategy for LA segmentation.

Harmonization	DS (%)	ST (%)	SP (%)	AUC (%)	ACC (%)	K (%)	VS (%)	HD
√	**76.83 (72.09–81.57)**	**77.23 (72.47–81.99)**	**99.19 (99.03–99.35)**	**97.14 (95.63–98.65)**	**98.26 (97.97–98.57)**	**75.92 (71.26–80.65)**	**91.80 (88.84–94.78)**	**38.02 (28.14–47.90)**
×	75.90 (70.67–81.12)	75.78 (70.14–81.42)	99.19 (99.01–99.37)	96.50 (94.90–98.10)	98.23 (97.90–98.56)	74.97 (69.76–80.18)	91.33 (88.47–94.18)	44.01 (29.07–58.94)

Bold values indicate the best performance.

**Table 4:** Ablation results for SWS classification.

Harmonization	Augmentation	ST (%)	SP (%)	ACC (%)	AUC (%)
×	×	87.31	98.75	95.58	97.38
√	×	85.95	98.94	95.65	96.05
×	√	84.45	98.75	94.86	98.35
**√**	**√**	**97.40**	**100.00**	**99.30**	**99.80**

Bold values indicate the best performance.

### 3.4 Comparison between human reader and model segmentation

**Figure 7:**
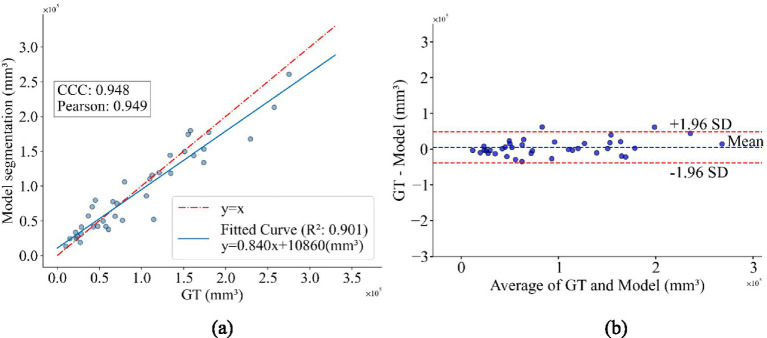
Agreement and consistency in LA volume between model segmentation and the ground truth (GT): **(a)** curve fit of LA volume between the model and the GT; **(b)** the scatter of the volume difference between the model and the GT. CCC: concordance correlation coefficient.

The volume of each LA marked by our model was calculated and compared to that annotated by the experienced neuro-radiologist, which served as the ground truth. The average difference in LA volume between our model and the ground truth was 4,607 mm^3^ (95% CI, −2,408 ~ 11,622 mm^3^), and the Pearson and concordance correlation coefficients were 0.949 and 0.948, respectively. Figure 7a shows the curve fit between the ground truth and the model, and Figure 7b shows the scatter of the volume difference.

**Figure 8:**
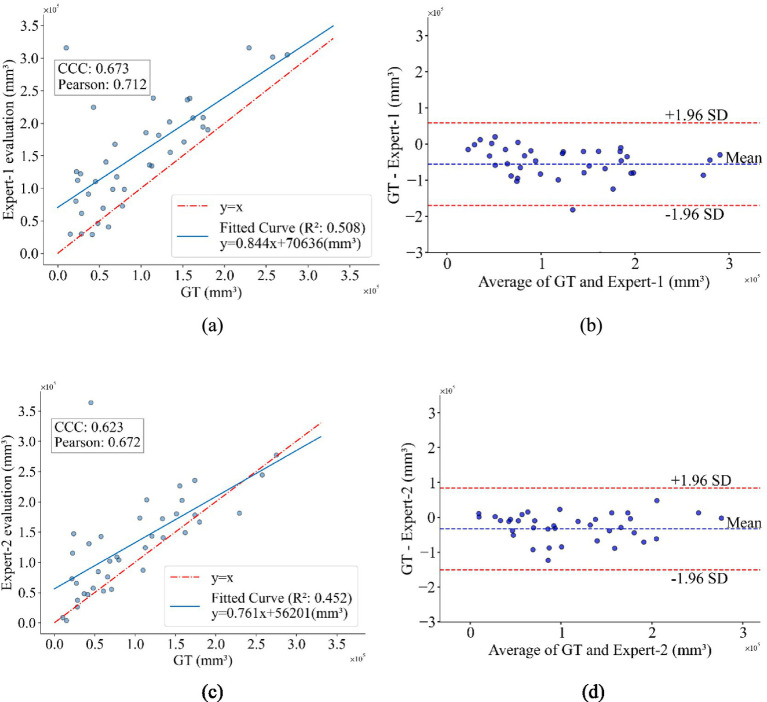
Agreement and consistency in LA volume between the two human readers (experts) and the ground truth (GT): **(a)** curve fit of LA volume between expert1 and the GT; **(b)** the scatter of the volume difference between reader1 and the GT; **(c)** curve fit of LA volume between expert2 and the GT; **(d)** the scatter of the volume difference between reader2 and the GT.

We also asked two less-experienced human readers to mark the LA independently using the same ITK-SNAP toolbox (Yushkevich et al., 2006). The voxel-level metrics of the two neuro-radiologists (respected to the ground truth) are reported in Table 2. The average volume difference between the two readers and the ground truth (annotated by the experienced neuro-radiologist) was −55,563 mm^3^ (95% CI, −74,072 ~ −37,054 mm^3^) and −33,091 mm^3^ (95% CI, −52,160 ~ −14,021 mm^3^), respectively. And the Pearson and concordance correlation coefficients were, respectively, 0.712 and 0.673 between reader1 and the ground truth, and 0.672 and 0.623 between reader2 and the ground truth. The curve fits and Bland–Altman plots were shown in Figure 8. It is clear that, for most of the metrics in both levels (voxel and volume), our deep learning model performs better than the two human readers, producing higher agreement and consistency with the ground truth.

In addition, the average inference time of the proposed model for one subject is 0.10 s, which is significantly reduced as compared with manual annotation (several minutes for one subject).

## 4 Discussion

LA in brain MRI images are the clinical manifestation of SWS (Schirmer, 1860; Sturge, 1969; Bodensteiner and Roach, 1999). Currently, detection of LA in MRI is carried out by manual visualization of the neuro-radiologists, which is time-consuming and experience-demanding. However, due to the rareness of this disease, neuro-radiologists with sufficient experience in SWS and LA labeling are lacking in many hospitals. A recent work has demonstrated the value of neuroimaging for recognizing atypical SWS presentations (Mengistie et al., 2025), and artificial intelligence (AI) has been suggested as a promising approach to improve clinical diagnosis in expertise-limited settings (Negussie et al., 2025). Our work is a pioneering study applying AI to detect and segment LA in brain MRI images. This proof-of-concept study shows promising performance in both LA segmentation and SWS diagnosis, consistent with experienced expert and much better than less-experienced neuro-radiologists.

In the present study, a fully automated method was proposed for LA detection and thus SWS diagnosis using a deep learning model that incorporated CNNs and Transformers in an nnU-Net framework. CNNs are widely used deep learning models and are very powerful in extracting local spatial features (Liu et al., 2019), and the Transformer models are particularly helpful to extract the global dependency of the spatial structures due to its self-attention mechanism (Dosovitskiy et al., 2021). The nnU-Net with encoding and decoding architecture has been suggested as the best framework for segmentation tasks (Isensee et al., 2020). Combining CNNs and Transformers in the nnU-Net framework can therefore effectively identify the enhanced leptomeningeal angiomatosis in the T1-weighted MRI. Our results show the proposed model produces better segmentation results as compared to simple UNet and nnU-Net models.

Furthermore, the proposed multi-task learning framework allows joint optimization of segmentation and classification tasks. This not only improves computational efficiency but also enhances feature representation by leveraging shared information between tasks. The strong agreement between the model and expert annotations, as well as its superior performance compared to less-experienced readers, suggests its potential clinical value.

The primary challenge of the present study is the limited sample size, e.g., only 40 patients collected in 15 years, which can hinder the application of deep learning methods. To overcome this challenge, the proposed deep learning model was first trained on the BraTs2021 dataset that consists of 2000 brain tumor MRI scans acquired from different institutions under standard clinical conditions, and then fine-tuned on our SWS dataset. We did not assume biological equivalence between gliomas and LA, but just utilized BraTS to initialize generic MRI feature representations before fine-tuning on the SWS dataset. Our results show that the pre-trained deep learning model produces better segmentation results (Dice 0.768) than that without pre-training (Dice 0.749). In addition, several strategies are employed to reduce the risk of overfitting, including data augmentation restricted to the training folds, weight decay regularization, and deep supervision during training.

Furthermore, the MRI data were acquired from multiple scanners over a long time span, providing substantial imaging heterogeneity that partially challenges model robustness. In this study, the proposed multi-scale harmonization strategy effectively reduces inter-scanner variability by normalizing intensity distributions across different imaging conditions. The improved segmentation and classification performance observed in our experiments demonstrates its effectiveness in enhancing model robustness. Note that the reference image was selected based on manual inspection of image quality and intensity distribution. In fact, selection of the reference image may influence the performance of the model. The sensitivity of reference-image selection was not evaluated in the present study and may be interesting direction for future studies.

Our results show the proposed deep learning model achieves similar segmentation performance to the ground truth (experienced neuro-radiologist), outperforming two human readers. These results suggest the presence of considerable disagreement and inconsistency in LA segmentation among different neuro-radiologists, and indicate the clinical potentials of deep-learning based LA segmentation, e.g., not only saving time but also improving the outcome of the clinical diagnosis.

Despite these promising results, several limitations should be noted. The relatively small sample size may still limit the generalizability, and the internal cross-validation cannot replace true external validation. In addition, only U-Net and nnU-Net were compared as baseline models. Comparison with more advanced models such as transformer-based model with external validation may boost the application of the developed model in real clinical application.

Furthermore, the quality of the ground truth LA labels can influence the performance of our deep learning model. In the present study, the labels were obtained from only one experienced neuro-radiologist due to limited expertise. Incorporating consensus labels of multi-experienced-neurologists during training may potentially improve model robustness. Moreover, the manual annotations represented LA-containing brain areas rather than exact abnormal vessel boundaries. This weak-label strategy may introduce boundary uncertainty and reduce voxel-level precision. Unsupervised or self-supervised learning relies less on the ground truth and has recently been proposed for less-reliable labels or missed labels (Ouyang et al., 2022; Liu et al., 2023). They may therefore produce improved LA segmentation and can be interested in future studies.

## 5 Conclusion

We have designed a multi-task deep learning framework for automatic segmentation of LA in T1-weighted MRI images and automatic diagnosis of SWS patients. By transferring a pre-trained model on a public dataset to our SWS dataset, the proposed automatic tool achieved similar segmentation performance to experienced neuro-radiologists and better performance than less-experienced neuro-radiologists. This proof-of-concept study may indicate promising potentials of deep learning method in clinical LA segmentation and SWS diagnosis, but limited by small sample size, single-center design, and weak labeling from only one neuro-radiologist.

## Data Availability

The raw data supporting the conclusions of this article will be made available by the authors under request.
